# Assessing fetal movements in pregnancy: A qualitative evidence synthesis of women’s views, perspectives and experiences

**DOI:** 10.1186/s12884-021-03667-y

**Published:** 2021-03-10

**Authors:** Valerie Smith, Kathryn Muldoon, Vivienne Brady, Hannah Delaney

**Affiliations:** 1 School of Nursing and Midwifery, University of Dublin Trinity College Dublin, Dublin, Ireland; 2 School of Nursing and Midwifery, National University of Ireland Galway, Galway, Ireland; 3 Health Research Board-Trials Methodology Research Network (HRB-TMRN), National University of Ireland Galway, Galway, Ireland

**Keywords:** Fetal movements, women’s’ experiences and views, Systematic review, Qualitative evidence synthesis

## Abstract

**Background:**

Raising awareness of the importance of fetal movements (FMs) and advising women on the appropriate action to take if they experience reduced FMs, is important for minimising or avoiding adverse perinatal outcomes. To gain insight and understanding of women’s perspectives of assessing FMs in pregnancy, we conducted a qualitative evidence synthesis.

**Methods:**

A qualitative evidence synthesis using thematic synthesis was conducted. Studies were eligible if they included pregnant women who were at least 20 weeks gestation and reported qualitative data from women on assessing FMs in pregnancy. MEDLINE, CINAHL, EMBASE, PsycINFO and Social Science Citation Index, from inception to July 2020, were searched. The methodological quality of included studies was assessed by at least two reviewers using an Evidence for Policy and Practice Information (EPPI)-Centre quality assessment tool. Data synthesis, using the Thomas and Harden framework, involved line by line coding of extracted data, establishing descriptive themes, and determining analytical themes. Confidence in the findings was assessed using GRADE CER-Qual.

**Results:**

Nine studies, involving 2193 women, were included in the review. The methodological quality of the studies was overall generally high. The synthesis revealed three dominant themes, and seven sub-themes that reflected women’s perspectives of assessing FMs in pregnancy. These were; 1) How women engage with FMs, with subthemes of informal engagement, formal engagement, and strategies to stimulate FMs; 2) *‘ … like a feather inside my belly’* - articulating and describing FMs, with sub-themes of sensations associated with FMs and timing and frequency of FMs; and 3) FMs and help/health seeking, with sub-themes of information sources and interacting with healthcare professionals. Confidence in the findings was either high or moderate, although two findings were rated low confidence and one very low.

**Conclusion:**

This qualitative evidence synthesis reveals that women informally engage with FMs during pregnancy. Women commonly adopt strategies to stimulate FMs when concerned. The use of the internet was a common source of obtaining information regarding FMs. Women require better support when contacting healthcare professionals about FMs. As only three of the nine included studies were exclusively qualitative in design, further qualitative studies exploring women’s perspective of assessing FMs in pregnancy are required.

**Supplementary Information:**

The online version contains supplementary material available at 10.1186/s12884-021-03667-y.

## Background

Fetal movements (FMs) in pregnancy have long been used as an indicator of fetal wellbeing. A reduction in or absence of FMs may indicate fetal compromise or death [1, 2]. The prevalence of women presenting with reduced FMs in pregnancy ranges from 4 to 23% across studies [3–5], with up to 55% of women experiencing a reduction in FMs in the days preceding an intrauterine death [6]. Raising awareness of the importance of FMs and advising women on the appropriate action to take if they experience reduced FMs is thus important for minimising or avoiding adverse perinatal outcomes.

Methods for assessing FMs in pregnancy are varied and can broadly be categorised as either subjective (passive or unstructured) or objective (active or structured) [7]. Subjective assessment relies on maternal perception and awareness of FMs rather than any formal or structured approach to monitoring FMs. Objective assessment, alternatively, uses a variety of tools for observing and/or recording FMs, for example; ‘kick charts’ or FM counting charts, and more recently, advanced technical methods such as multisensor magnetocardiographic recordings, mobile applications and abdominal sensors [8–10]. Evidence for the effectiveness of objective FM monitoring methods for improving perinatal outcome however is lacking. A recent systematic review and meta-analysis comparing perinatal outcomes in women instructed to count their FMs compared to no counting instructions found no difference between the groups in the incidence of perinatal death or morbidity [11]. The large AFFIRM trial, however, contributed most of the data to the meta-analyses in this review. AFFIRM was a multicentre stepped wedge cluster trial that evaluated a package of care involving raising awareness of the importance of reduced FM in pregnancy combined with a structured approach to fetal assessment and expedited birth where the benefits were likely to outweigh the risks [12]. The trial involved 409,175 pregnancies of which 227,860 births occurred during the intervention period. Although the results showed a decrease in stillbirth at or after 24 weeks’ gestation, from 4.40 per 1000 births in the usual care group to 4.06 in the intervention group, and in perinatal mortality, from 6.38 and 5.77 per 1000 births in the usual care and intervention groups respectively, the differences between the groups was not statistically significant [12].

Mindfetalness, an alternative approach to assessing FMs which involves women focusing on the characteristics of FMs, such as strength and frequency, rather than counting each FM, has also been evaluated in a large cluster trial (*n* = 39,865 women) in Sweden. Although the trial was powered to detect a difference in Apgar scores (< 7 at 5 min of age) as the primary endpoint, the trial also measured perinatal death within 27 days of birth. No difference between the intervention and control groups was found (2 versus 5 deaths respectively, *p* = 0.27), although spontaneous onset of labour was higher in the Mindfetalness group, and caesarean section rates were lower [13]. Variation in guidance, recommendations and practices for assessing FMs in pregnancy thus remains [14–16], and women continue to receive varied levels of information and advice from healthcare professionals regarding FM assessment [17, 18].

The impact of FM assessment on pregnant women requires consideration in the context of clinical effectiveness and subjective affect (e.g, maternal acceptability, worry/concern, satisfaction, etc.). For this reason, we conducted a qualitative evidence synthesis to gain insight and understanding of pregnant women’s perspectives of assessing their FMs during pregnancy. Thomas and Harden’s thematic synthesis method was used to guide the synthesis of the data [19]. The study was prospectively registered with the International Prospective Register of Systematic Reviews (PROSPERO), available at: https://www.crd.york.ac.uk/prospero/display_record.php?ID=CRD42019144590 and adheres to the Enhancing Transparency in Reporting the synthesis of Qualitative research (ENTREQ) guidance ([20]; Additional file 1).

## Methods

### Inclusion criteria

Studies were eligible for inclusion if they met the following participants, exposure, outcomes and study type criteria;

- *Participants:* Pregnant women (at the time of participating in a study) of any parity or risk status who have reached gestational age of at least 20 weeks;
- *Exposure:* Expression of views, perceptions or experiences of assessing FMs during pregnancy. These expressions could be drawn from maternal subjective assessments or reports related to awareness of FMs, or from more structured methods of monitoring such as the use of ‘kick’ charts, or technical aids;
- *Outcomes*: Inductive dominant themes representative of women’s views, experiences, and perceptions of assessing FMs in pregnancy;
- *Study type*: Studies providing qualitative data of women’s perspectives of FMs in pregnancy. Qualitative studies of any design were eligible. Studies of mixed methods design, where qualitative data could be extracted separately, were included. Survey designs with open-ended questions that provided qualitative data were also considered for inclusion.

### Search strategy

To identify relevant records, a search of the following electronic databases, from their date of inception to September 2019, and updated again in July 2020, was performed: MEDLINE, CINAHL, EMBASE, PsycINFO and Social Science Citation Index (via the Web of Science). Search terms used to guide the search centred on fetal movement terms, combined with the Boolean operands ‘OR’ and ‘AND’ as appropriate, and adapted across the databases. Synonyms (e.g, fetal OR foetal) were also considered prior to implementing the search; for example using EMBASE, the search strategy was (MM “Fetal Well-Being”) OR (MM “Fetal Movement”) OR “fetal movement OR fetal movements OR fetal activity OR foetal movement OR foetal movements OR foetal activity OR fetal wellbeing”. The electronic database searches were supplemented with searches of grey literature websites (Open Grey, http://www.opengrey.eu/) and proceedings of the International Confederation of Midwives Triennial Conference (2017). The reference lists of retrieved full-text papers were searched also for any additional papers that might not have been captured by the database searches. Searches were not limited on language; however, due to an inability to translate non-English language texts it was necessary to select studies published in English only. Searching all languages helped us to identify possible language bias by highlighting the number of non-English papers that might have been relevant. Studies involving the same study sample reported across two or more publications were included only where these records reported different findings to each other. Searching and selection of each citation was undertaken independently by at least two reviewers (VS, KM and HD).

### Quality assessment

The methodological quality of the included studies was assessed using an appraisal tool that was developed by the Evidence for Policy and Practice Information (EPPI) and Co-ordinating Centre for use originally in a systematic review of healthy eating in children [21]. The tool consists of 12 quality appraisal criteria that focus on the quality of a study’s methods and the study report. Each included study was assessed independently by at least two reviewers (VS, KM and HD) on the extent to which each quality criterion was met. Quality assessments by pairs of reviewers were then compared, and agreed, or if required, a third reviewer was consulted until consensus was reached. Considering that even poorly conducted or reported qualitative studies can provide important information on ‘views’, data from all studies, irrespective of quality, were extracted and used for synthesis purposes.

### Data extraction and synthesis

Data were extracted from each included study independently by a pair of reviewers (VS, KM and HD) using a purposively designed data extraction form (Additional file 2). The following information was extracted; aim of study, study design, description of participants and setting, method of data collection and analysis, and findings related to women’s views, perceptions, and experiences of assessing their baby’s movements during pregnancy. Guided by Thomas and Harden’s framework [19], synthesis of the data involved three stages: i) line by line coding of extracted data, ii) development of descriptive themes and, iii) generating analytical themes. Similarities and differences between codes were identified and grouped to generate descriptive themes. Analytical themes and sub-themes were generated through additional synthesis, reflection, discussion and iteration. To add rigor to the synthesis process, stages i) and ii) were undertaken independently by two reviewers. A process of line by line coding of each of the included study’s data was undertaken separately by at least two reviewers (VS, HD and VB), and descriptive themes were identified. The review team subsequently met to compare codes, review descriptive themes, and refine accordingly, based on iteration, discussion and consensus. Stage iii) was undertaken by one reviewer (HD), and corroborated by a second reviewer (VS).

### Assessment of confidence in the review findings; GRADE-CERQual

**Fig. 1:**
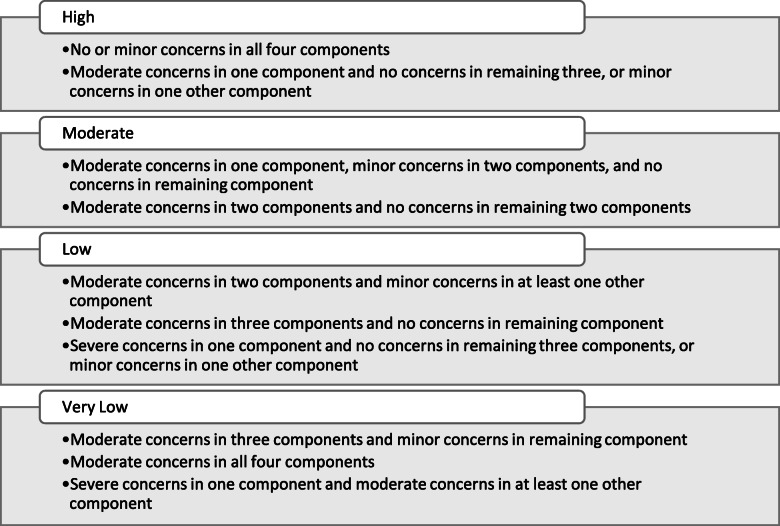
GRADE-CERQual downgrading criteria

To assess levels of confidence in the review findings, we applied the Grading of Recommendations Assessment, Development and Evaluation-Confidence in the Evidence from Reviews of Qualitative research (GRADE-CERQual) [22–27]. Using GRADE-CERQual, each discrete review finding was assessed under four components. These were: the methodological limitations of the studies contributing to the finding, the coherence of the finding, the adequacy of data contributing to the finding and the relevance of the contributory studies to the review question. Following these assessments, an overall assessment of confidence in each finding was made, and categorised as High, Moderate, Low or Very Low confidence [22]. As equal weighting is attached to each of the four components, we established a priori downgrading criteria as illustrated in Fig. 1.

As recommended, the GRADE-CERQual assessments were performed by a pair of reviewers (VS and HD) with final judgements based on discussions and consensus [23]. All judgements were based on an initial assumption of ‘High confidence’ in all findings, and then downgraded accordingly.

## Results

### Search and selection

**Fig. 2:**
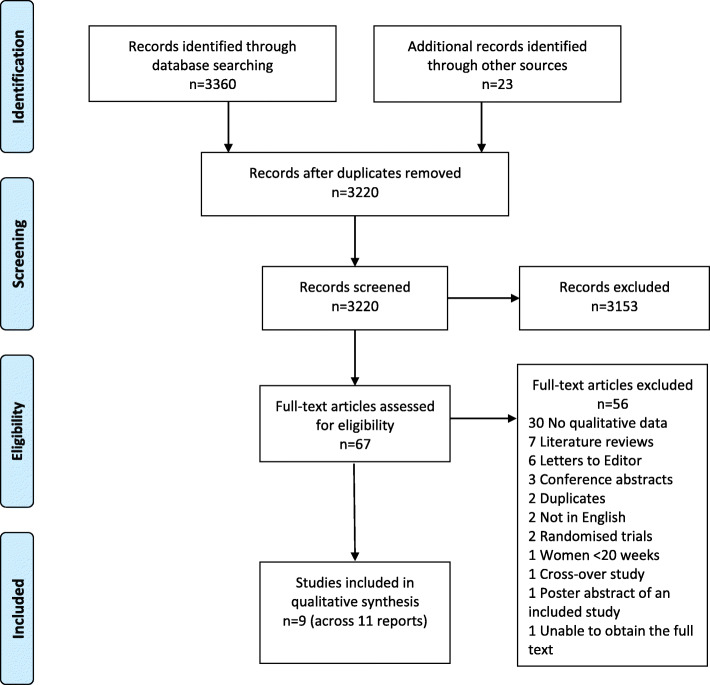
Search and Selection Flow Diagram

The results of the search yielded 3360 citations from the electronic database sources and a further 23 from searching of other sources. Of these 3383 citations, 163 were identified as duplicates and removed leaving 3220, which were assessed on title and abstract. A further 3153 were excluded at title and abstract screening as they were clearly not eligible. The remaining 67 citations were screened at full-text level. Fifty-six of these were subsequently excluded for the following reasons; 28 had no qualitative data specifically on views, experiences or perceptions of assessing FMs, seven were literature reviews, six were letters to journal Editors, four were conference abstracts with insufficient qualitative data to include, two were identified as further duplicate reports, two were not in English (reflecting limited language bias), two were randomised trials, one included women at less than 20 weeks of pregnancy, one was a cross-over study with insufficient qualitative data to include, one was a poster abstract of an included study, one was a conference abstract of an included study and, for one, we were unable to obtain the full text to accurately assess eligibility. The references, and exclusion reasons for these 56 excluded studies are provided in Additional file 3. This resulted in the inclusion of nine studies across 11 publications [28–38]. Figure 2 illustrates the search and selection process.

### Description of included studies

Table 1 presents the summary characteristics of the included studies. Three studies (four reports) were conducted in Sweden [28, 32, 33, 36], three in Australia [34, 35, 37], two in the UK [31, 38] and one (two reports) in New Zealand [29, 30]. The majority of the studies (*n* = 6) were conducted from 2011 onwards, with one conducted in 1986 [31], and for two, the study dates were not provided [29, 30, 36]. Data collection involved the use of questionnaires with open-ended response options in six studies [28, 31–37] and interviews in the remaining three studies [29, 30, 36, 38]. In total, 2193 multiparous and primiparous women participated in the nine included studies (Table 1).

**Table 1:** Summary characteristics of included studies

Reference	Aim	Year study conducted	Description of participants	Description of setting	Data collection method	Data analysis method
Akselsson 2017 [28]	To explore women’s attitudes, experiences and compliance concerning the practice of Mindfetalness in late pregnancy	15 Feb −7 Jul 2016	104 women, 17–42 years of age, 28–32 weeks of pregnancy	Three maternity clinics in Stockholm, Sweden	Midwife administered questionnaire	Qualitative manifest content analysis
Bradford & Maude 2014 [29]; Bradford & Maude 2018 [30]	To explore normal fetal activity in the third trimester as perceived by pregnant women themselves	Not stated	19 low-risk nulliparous women, 19–34 years and ≥ 28 week’s gestation	Five community-based midwifery practices in a provincial city in the North Island of New Zealand	Interviews conducted in the third trimester at two time-points; 28–32 weeks and 37–41 weeks	Qualitative content analysis
Draper 1986 [31]	To report on the views of women on filling in fetal movement charts during pregnancy	1982 and 1983	132 women, 27–37 weeks gestation	Community antenatal clinic in Cambridge	Interviews and postal questionnaire	Not stated
Linde 2016 [32]; Linde 2017 [33]	To examine how women, who consulted health care due to RFM, describe how the baby had moved less or differently, and to explore why women decide to consult health care due to RFM and investigate reasons for delaying a consultation	Jan-Dec 2014	960 women of median age 32 years and ≥ 28 week’s gestation	Seven delivery wards in Stockholm, Sweden	Questionnaire with open-ended response options	Modified content analysis
McArdle 2015 [34]	To investigate sources pregnant women used to acquire information about FMs and their preferences for receiving this information	Dec 2011-Mar 2012	526 women of mean age 30.5 years and ≥ 32 week’s gestation	Antenatal clinic of a large metropolitan maternity hospital, Australia	Questionnaire with open-ended questions	Content analysis
Pollock 2020 [35]	To explore the ANC experiences of Australian mothers who had recently had a live birth to determine their knowledge of FMs	May-Oct 2017.	391 women, > 18 years of age who had given birth to a live baby within the last ten years	Australia	Online survey with open ended questions	Summative content analysis
Rådestad & Lindgren 2012 [36]	To explore women’s perceptions of FMs in full-term pregnancy	2011	40 women, 23–40 years old, between 37 + 2 and 41 + 5 week’s gestation	One antenatal clinic in the capital of Sweden	Interviews	Thematic analysis
Raynes-Greenow*,* 2013 [37]	To examine maternal perception of normal FMs, and to describe FM advice in a routine antenatal care setting	Not stated	156 women ≥28 weeks gestation of mean age 32 years	A major metropolitan tertiary referral hospital in Sydney, Australia	Self-administered questionnaire with open-ended questions	Thematic analysis
Smyth 2016 [38]	To explore what triggers women to access health care after experiencing RFM and conversely what stops them	Aug 2012-Feb 2013	21 women of mean age 27 years, and gestation at time of RFM 32 weeks	Large teaching hospital in the North-West of England	Semi-structured interviews	Framework analysis

*ANC* Antenatal Clinic, *FM* Fetal Movement, *RFM* Reduced Fetal Movement

### Quality assessment

Table 2 presents the results of the quality assessment. None of the nine included studies met all 12 quality criteria. Two studies met 11 of the 12 criteria, with both not meeting the criterion of actively involving the participants in the design and conduct of the study [29, 30, 38]. Three studies met 10 of the 12 criteria [32, 33, 36, 37]. Two studies respectively met nine and eight of the 12 quality criteria [35, 36]. Of the remaining two studies, one met five [28] and the second met three of the 12 criteria only [31].

**Table 2:** Methodological quality of included studies

**Author and year**	**Quality criteria**^**a**^ **met**	A: Aims and objectives clearly reported B: Context of the research adequately described C: Sample and sampling methods described D: Data collection methods described E: Data analysis methods adequately described F: Reliable data collection tools established G: Valid data collection tools H: Reliable data analysis I: Valid of the data analysis J: Appropriate data collection methods used to allow for expression of views K: Used the appropriate methods for ensuring the analysis was grounded in the views L: Actively involved the participants in the design and conduct of the study
Akselsson 2017 [28]	A, B, D, E, K	
Bradford & Maude 2014 [29]; 2018 [30]	A, B, C, D, E, F, G, H, I, J, K	
Draper 1986 [31]	B, F, G	
Linde 2016 [32]; 2017 [33]	A, B, C, D, E, F, G, H, I, K	
McArdle 2015 [34]	A, B, C, D, E, F, G, H, I	
Pollock 2020 [35]	A, B, C, D, E, G, H, I	
Rådestad & Lindgren 2012 [36]	A, B, C, D, E, F, G, H, J, K	
Raynes-Greenow 2013 [37]	A, B, C, D, E, F, G, H, I, K	
Smyth 2016 [38]	A, B, C, D, E, F, G, H, I, J, K	

^a^Letters indicate that the corresponding quality criterion was met in the study report

### Findings

Three dominant analytical themes with seven subthemes emerged from the thematic synthesis. Additional file 4 provides an audit trail of the synthesis process from identifying codes, to descriptive themes and finally analytical themes. Table 3 illustrates the studies that contributed data to each of these themes/sub-themes.

**Table 3:** Studies contributing data to themes/subthemes

	How women engage with FMs			Articulating and describing FMs		FMs and help/health seeking	
	Informal engagement with FMs	Formal engagement with FMs	Strategies to stimulate FMs	Sensations associated with FMs	Timing and frequency of FMs	Information sources	Interactions withHCPs
Akselsson 2017 [28]	✓	✓					
Bradford & Maude 2014 [29]	✓			✓	✓		
Bradford & Maude 2018 [30]	✓		✓	✓		✓	✓
Draper 1986 [31]	✓	✓			✓		✓
Linde 2016 [32]	✓		✓		✓		
Linde 2017 [33]	✓		✓		✓		✓
McArdle 2015 [34]	✓					✓	
Pollock 2020 [35]	✓	✓	✓	✓	✓	✓	✓
Rådestad & Lindgren 2012 [36]	✓			✓			
Raynes-Greenow 2013 [37]	✓		✓	✓	✓	✓	✓
Smyth 2016 [38]	✓		✓			✓	✓

*FMs* Fetal movements, *HCPs* Healthcare professionals

#### Theme 1: how women engage with FMs

All nine included studies contributed data related to women’s engagement with FMs in pregnancy. Three subthemes were identified which encapsulate these narratives. These were informal engagement with FMs, formal engagement with FMs, and strategies used to stimulate FMs.

### Informal engagement with FMs

All nine studies referred to how women engaged subconsciously with or monitored their FMs in an informal way. Factors that women perceived to impact on FMs were varied and included their own position or their baby’s position, time of day, and their hunger/eating patterns [28–30, 37]. Although FMs varied throughout the day and from hour to hour, in general, women described experiencing increased fetal activity more often in the evenings [30, 31] and before meals [29, 37] and decreased fetal activity after meals [30]. Women described that drinking coffee, sweet drinks or cold water also had the effect of increasing FMs [32, 33, 37].

‘*I lie on my back instead of on the side, otherwise the baby protests because she/he doesn’t like the side* ([29], p.3).

*‘ … she gets very excited before dinner time’* ([30], p.4).

and, referring to feeling hungry, one woman describes how her baby gets ‘ … ..*really wriggly and really squirmy’* but *‘feels a lot more comfortable after I’ve eaten’* ([29], p.4).

Women associated FMs with good fetal health. Regular patterns of FMs were considered reassuring and a way of feeling ‘*connected*’ to their baby. A pattern of movement was an expectation of healthy fetal behaviour, although this pattern was recognised as being individual for each woman. These individual patterns were also a point of reference for women in identifying reduced FMs [31–33];

*‘The baby has not moved at the times that she had moved earlier, following the pattern that she had previously … ..the movements felt weaker the past two days compared to before’* ([32], p.4).

Women also reported struggling to identify a pattern which made FM monitoring more difficult and interfered with women relaying information about FMs to clinicians. Expressed expectations of frequency and quantity of FMs also varied, ranging from a few times each day, to four per hour or at least 10 per hour. Women’s narratives also highlighted uncertainty around what they should expect of FMs;

*‘I would like to know the normal number of movements for babies of different gestations’* ([34], p.575).

*‘I believe its 4* [movements] *per hour on average, maybe?’* ([35], p.81).

Women subconsciously engaged with and monitored FMs from the beginning of their pregnancy. Some experienced doubt and uncertainty when attempting to identify first movements, finding it difficult to distinguish between actual FMs and other sensations, until a pattern or more consistent sensations became established;

*‘It was just one little tiny movement and I wasn’t sure if it was, but then movements after that felt the same’* ([30], p.289).

For women, identifying their first FMs made their pregnancy and baby feel real, although initial sensations could be ‘*a little unpleasant’* ([36], p.114). Informal monitoring of FMs also acted as a mechanism of communication between the mother and her baby [28–30]. Women became more aware of the baby ‘*as an individual’* and felt more ‘*connected’*; when FMs were visible and palpable, this experience of FMs could then be shared with family members; *‘my husband is also with me and listens, he has his hands on my tummy during this time’* ([28], p.4).

### Formal engagement with FMs

Three studies provided data on formally assessing FMs [28, 31, 35]. In one of the studies, women, in practising Mindfetalness, monitored their FMs in a structured way by focusing on the intensity and character of their FMs, without necessarily counting them [28]. In the other two studies the use of the Cardiff count-to-10 was explored [31] and women’s comments on tracking FMs were collected [35], with women recounting that they would take time out to count FMs and the importance of this.

For some women, formally assessing FMs caused worry [28, 31]. Although the exact nature of this worry was not specified by all women, they did report feeling anxious until the required number of kicks had been counted and that focusing on FMs in such a structured format could cause more worry. Others expressed doubt about identifying what specifically constitutes a ‘*kick*’ [31]. Formally engaging in FM recording was also considered an inconvenience by some women, mainly in terms of lack of time, losing count, and forgetting to complete the FM chart, particularly towards the end of pregnancy. Other women questioned the value of using a ‘*kick*’ chart, suggesting that they would notice if their babies’ movements stopped and that a chart was not necessary for this [31]. The value of recording FMs formally is further questioned by one woman’s comment where, rather than use a chart, she

‘*would have preferred to have been told to notice and report changes in her baby’s movements*’ ([31], p.336).

In contrast, women also felt that monitoring their babies’ FMs formally was very important so as ‘*to gain an understanding over time of what is ‘normal’ for you and your baby’* ([34], p.33). These women were happy to complete a FM chart, and did not view it as an inconvenience [31]. Formal FM monitoring provided women with reassurances that their babies were kicking and that this meant that their baby was well. Some women stated that they felt more confident and less worried about FMs when a method of formally assessing them was used. This was especially so for women using Mindfetalness, where the characteristics of FMs, such as intensity and pattern, are noted;

*‘I practice the method more when I get worried about fetal movements. Now, I’m not as worried as before”* ([28], p.4)

### Strategies to stimulate FMs

Women commonly adopt strategies to elicit FMs when they were experiencing altered or reduced FMs [30, 32–34, 37, 38]. For instance; drinking a sugary, citrus or cold drink to ‘*shock the baby and wake it up*’ [37], physically moving the body or compressing it by ‘*rubbing or prodding the belly*’ [30], or ‘*pulling and nudging the tummy*’ [32]. Other strategies adopted by women included having a warm bath, placing hands on the abdomen, and lying down. Generally, across the studies, women reflected that if these strategies did not elicit FMs, further care from a healthcare professional was required;

*‘ … ..try to encourage movements, stand up, move around, have a sugar, citrus drink. If still no movements/reduced movements, go to hospital’* ([35], p, 81).

#### Theme 2: ‘ … like a feather inside my belly’ – articulating and describing FMs

The theme of articulating and describing FMs is illustrated in two sub-themes. These are sensations associated with FMs and timing and frequency of FMs.

### Sensations associated with FMs

Women’s descriptions and sensations of FMs differed at different gestational ages. Characteristics of the first fetal sensations included being ‘*very soft … like a puff of air … very gentle*’ [37] and were described in terms of feeling like a small ‘*knock,’ ‘dink’*, ‘*hiccup*’ or ‘*jolts*’ [30, 37]. One woman, in describing these early FMs commented that it took time for her to become accustomed to the nature of her baby’s FMs;

*‘ … it felt so jerky and I couldn’t imagine what it was doing, but now I have got used to feeling that way*’ ([36], p.114).

Women’s descriptions of FMs changed as pregnancy progressed. Descriptions of FMs at the start of the third trimester were varied [29, 30, 36, 37], with women describing more specific limb movements that were sometimes visible on the skin;

‘*you can sometimes see the actual skin moving. I can’t tell what it is; like an elbow, knee or foot, but just seeing the skin move*’ ([30], p.290)

These limb movements were described as ‘*punchy*’, with whole body movements described using a variety of terms from ‘*smooth*’ or soft ‘*wriggling’* and ‘*tapping’* movements to stronger ‘*kicking’* or ‘*swooping’* movements [30, 37]. As the baby reached term, women described movements as becoming less varied, slower, and stronger [29, 30, 36, 37];

‘*like a film in slow motion … there is a lot of power, but everything is going slowly, gliding along. I imagine a wrestling match, maybe in slow motion. You see lots of power, but things move slowly’* ([36], p.114)

Women interpreted these slower, stronger and altered FMs as the baby having less space as the end of pregnancy approached ‘*as the baby gets bigger’* and *‘has less room to move’* ([37], p.5) although there appeared to be some confusion amongst women as to expectations of FMs towards term; *‘Close to birth … ..movements will less a bit’* [35] and *‘slow down’* because there is *‘less room’* [35, 37] versus ‘*movements should not slow down towards the end of pregnancy even if the baby has less room’* ([35], p.5).

### Timing and frequency of FMs

Variations in frequency and timing of FMs was a common experience for women, with some experiencing regular FMs throughout the day [29, 30, 37], while others experienced less movement during the day, more commonly experiencing FMs in the evening [30, 31]. Expectations as to when first FMs should be felt varied between 12 and 19 weeks and 17–20 weeks, although many women (approx, 25% in one study [37]) report feeling their first FMs after 20 weeks’ gestation [35, 37]. Women associated unusual or changed FMs with changes in the frequency of FMs, absence of FMs, changes in the sensation of FMs, FMs not occurring at the usual time, occurring less often or becoming weaker and non-specific [32, 37]. Reduced or an absence of movement, including concerns for these, was generally framed in the context of time;

‘*I haven't felt any kicking for about 12 hours*’ ([32], p.3).

*‘When the activity had decreased and had not gone in the right direction after 2 days’* ([33], p.378).

#### Theme 3: FMs and help/health seeking

Women provided various accounts related to help and health seeking behaviours and views with respect to FMs in pregnancy. These perspectives are reflected in the sub-themes of information sources and interacting with healthcare professionals.

### Information sources

Women reported accessing multiple information sources on FMs including, healthcare professionals, antenatal classes, books, the internet, family and friends [30, 34, 35, 37, 38]. There were preferences for receiving information on FMs from healthcare professionals, especially midwives (82% of 526 women in one study [34]), and particularly in the format of printed documentation such as a pamphlet or hand-out, rather than verbal information [34, 35]. The main reason for this was that printed information could be easily referred to if needed;

*‘a hand-out to read throughout pregnancy, so we can refresh our cloudy heads*’ ([34], p.57).

Women also indicated a desire for specific information about monitoring FMs, such as information on movement counts/types/changes and when to seek advice [34, 35, 37]; although a preference for more general information about health and wellbeing rather than information that was specific to FMs only was also expressed ‘*so as not to distress or cause too much anxiety*’ ([35], p.82).

Women commonly sought informal information about FMs from their friends or family [30, 34, 38], and for some women they relied on this information in advance of or as an alternative to contacting their midwife [30]. Others compared experiences with their peers and consulted family members who had experienced pregnancy previously [38]. The internet was a common source of information for women on FMs [34, 35, 37, 38] often as the first source of advice or instead of consulting a healthcare professional as it is ‘*more accessible’* [38]. Online forums were described as helpful, although they could cause worry too [38], which might explain why women expressed preferences for trusted websites such as ‘NHS direct’ and sought direction to trusted websites from their healthcare providers [34].

### Interacting with healthcare professionals

Six of the included studies described women’s interactions with healthcare professionals about FMs [30, 31, 33, 35, 37, 38]. A decrease in FMs was generally perceived as a cause for concern that warranted help from a healthcare professional. Others distinguished between a reduction in FMs, and no movement at all which was a cause for greater concern;

*‘ … as long as she moved then I consider that to be okay. I think if it’s been a couple of days and they’ve not moved or a full day then it’s something to worry about*’ ([38], p.3).

Reasons for contacting healthcare professionals due to a decrease or change in FMs included a defined period of time had passed with decreased or altered movement, although this varied from a few hours to a number of days, if the worry became unmanageable, when women experienced a fear of fetal loss, and when strategies to stimulate movements were unsuccessful [33, 34, 38]. Barriers to contacting healthcare professionals were mostly related to doubts or fears of being perceived in a particular way. Concerns experienced by women included fears that they would not be taken seriously, not listened to, or that they may be viewed as *‘hysterical’*, ‘*overly anxious’*, or *‘being a hypochondriac’* [35, 37, 38] with fears often based on previous negative interactions;

*‘I was made to feel uneducated and overly anxious, and at times I agonised whether to take my concerns to the professionals or just ‘Dr, Google’ … to save face and stress*’ ([35], p.80).

Other barriers to contacting healthcare professionals included feelings of uneasiness that they were taking up the healthcare professionals time unnecessarily and concerns that they would be induced or be perceived as trying to get induced [33, 35, 38]. Contrary to this, healthcare professionals were explicit on what to do should women experience reduced or altered FMs and women responded actively to this advice;

*‘My midwife at antenatal care has told me clearly that I should call the birth clinic if I experience decreased fetal movements’* ([33], p.378).

*‘It was the midwife when I saw her … ..and straight away she was like, you need to ring triage, we need to get it checked out. So that what prompted me to call in’* ([38], p.5)

Advice from healthcare professionals on monitoring FMs and on what to do if they were concerned about FMs varied. This ranged from making contact with a healthcare provider if there was any reduction or change in FMs, not to worry as long as there were some FMs everyday regardless of quantity, specific advice on expected frequency and quantity of FMs, and little or no advice at all [32, 34, 37, 38];

*“During visits I have only been asked if the baby has moved – I reply yes and the conversation ends’* ([34], p.57).

#### Confidence in the review’s findings – CERQual

Overall, confidence in the review’s findings was either high or moderate, with two of 16 discrete findings receiving a low confidence rating, and one only receiving a very low confidence rating. The finding rated very low confidence related to the formal assessment of FMs, and the resulting worry and anxiety, as well as reassurances that can come from this. This finding was downgraded to very low because the majority of the contributing data was from three studies, two of which met five or less of the 12 quality criteria (serious concerns), the data supporting the finding was varied (moderate concerns) and all of the data came from open-ended response options in surveys (moderate concerns). The two findings rated low confidence related to women’s expectations for when first FMs might be felt and that women commonly experience FMs in the evenings. Table 4 provides the summary results of the CERQual assessments. The Evidence Profile and rationale for judgements in each of the four components and overall confidence rating for each discrete finding, is provided in Additional file 5.

**Table 4:** Summary results of CERQual assessments

Finding	Contributing reports	Methodological limitations	Coherence	Adequacy	Relevance	Overall Confidence
**Analytical theme:** ***How women engage with FMs***						
Women identified perceived factors that impact FMs such as; mother’s position, time of day, and mother’s hunger/eating patterns	28–30,37	No or very minor concerns	Minor concerns	Minor concerns	Moderate concerns	**Moderate**
Women associated FMs with health; regular, individualised patterns of FMs were viewed as reassuring and altered patterns as a cause for concern	31–33,34,35	No or very minor concerns	No or very minor concerns	Minor concerns	Moderate concerns	**High**
Informal monitoring of FMs acted as a mechanism of communication between mother and baby	28–30	No or very minor concerns	No or very minor concerns	Minor concerns	Minor concerns	**High**
Formal engagement with and assessment of FMs can cause worry and anxiety, but was also considered important, providing reassurances that the baby was well	28,31,35	Serious concerns	Moderate concerns	Moderate concerns	Minor concerns	**Very Low**
When women were experiencing reduced or altered FMs, they adopted a variety of strategies to elicit movement	30,32-34,37,38	No or very minor concerns	No or very minor concerns	Moderate concerns	Moderate concerns	**Moderate**
**Analytical theme:** ***Articulating and describing fetal movements***						
Women’s descriptions and sensations of FMs differed at different gestational ages with changes in FMs noted as pregnancy progressed	29–30,35–37	No or very minor concerns	Minor concerns	Minor concerns	Minor concerns	**High**
Women’s expectation of the timing of first FMs and the frequency they experienced FMs throughout the day were varied	29,30,35,37	Minor concerns	No or very minor concerns	Moderate concerns	Moderate concerns	**Low**
Women commonly experienced increased FMs in the evening and before mealtimes	30,31	Moderate concerns	No or very minor concerns	Moderate concerns	Minor concerns	**Low**
Women associated unusual or changed FMs with changes in frequency or absence of FMs, or changes in the sensation of FMs	32,33,37	No or very minor concerns	No or very minor concerns	Moderate concerns	Moderate concerns	**Moderate**
**Analytical theme:** ***Fetal movements and help/health seeking***						
Women accessed multiple information sources on FMs including; healthcare professionals, antenatal classes, books, the internet, and family and friends	30,34,35,37,38	No or very minor concerns	No or very minor concerns	Minor concerns	Minor concerns	**High**
There were preferences towards receiving FM information particularly in the format of printed documentation such as a pamphlet or hand-out, although preferences for the types of information were mixed	34,35,37	No or very minor concerns	Minor concerns	Minor concerns	Moderate concerns	**Moderate**
The internet was a common source of information often ahead of consulting a healthcare professional	34,35,37,38	No or very minor concerns	No or very minor concerns	Minor concerns	Moderate concerns	**High**
A decrease in FM was generally perceived as a cause for concern that warranted help from a healthcare professional	30,31,33,35,37,38	No or very minor concerns	Minor concerns	Moderate concerns	Minor concerns	**Moderate**
Reasons for contacting healthcare professionals due to a decrease or change in FMs included; if a defined period of time had passed, if the worry became unmanageable, fear of fetal loss, unsuccessful strategies to stimulate FMs	33,34,38	No or very minor concerns	No or very minor concerns	Minor concerns	Minor concerns	**High**
Barriers to contacting healthcare professionals were mostly related to doubt or fear of being perceived a particular way, not being listened to, wasting healthcare professionals’ time	33,35,37,38	No or very minor concerns	Minor concerns	Moderate concerns	Minor concerns	**High**
The advice offered by healthcare professionals to women on monitoring FMs and on what to do if they were concerned about FMs varied	32–34,37,38	No or very minor concerns	No or very minor concerns	Moderate concerns	Moderate concerns	**Moderate**

## Discussion

This synthesis of evidence from nine studies presented across three themes and seven sub-themes, provides understanding and insight from the perspectives of women on FM assessment in pregnancy. Having knowledge and awareness of these findings is important to maternity care providers so that appropriate and optimal discussions surrounding FMs in pregnancy can take place. Having insight of women’s perspectives around FMs is also essential for providing holistic maternity care whereby women’s views and experience as evidence is valued alongside objective empirical methods.

Women in pregnancy commonly engage with assessing FMs, whether subjectively through perception and awareness, or objectively by using more formal monitoring methods. Women’s narratives highlight the individualistic nature of FMs where patterns and characteristics vary throughout the day, and as pregnancy progresses. Women, reassuringly, appear to recognise this, and, in connecting with their baby, come to understand their own baby’s individual FM pattern, and how changes to this might be a cause for concern.

Views and experiences on methods for formally assessing FMs, were mixed. Women viewed ‘kick charts’ for example as an inconvenience, and they caused worry for some women. Contrastingly, several women were happy to complete kick charts, and felt that they helped them get to know their baby’s pattern of movement. This finding should be considered in the context of limited available evidence on the effectiveness of objective FM assessment methods in reducing perinatal adversity [11–13] balanced with evidence of the benefits for increased maternal-fetal attachment [14–16]. This is coupled with the evidence supporting the use of a more structured approach to FM assessment in assisting women get to know their baby’s individual FM pattern. This finding indicates that healthcare providers should be sufficiently knowledgeable to discuss the various methods of FM assessment (both formal and informal) with women, and how these methods might be used depending on women’s individual choice and expressed preferences for approaches to FM assessment in pregnancy.

This synthesis also offered insight into the strategies women adopt for stimulating FMs. The effectiveness of these strategies however, is largely unknown. For example, taking sugary drinks for stimulating FMs appeared as a common misconception in our synthesis. Evidence to support this practice is lacking, or at best conflicting, and women should be advised accordingly. Studies that explored the effect of increased maternal blood glucose levels on FMs support the hypothesis that raised maternal blood glucose concentrations can result in increasing FMs [39], although results from other studies refute this, demonstrating no evidence of effect [40, 41]. Although, the findings of this evidence synthesis highlight that women will seek professional care if a strategy does not elicit FMs, knowing that women spend time adopting strategies is insightful for healthcare providers as it might indicate women will delay seeking care in preference for spending time on such strategies. Ultimately, all women should be encouraged and supported to report any decrease or cessation of FMs to their maternity care provider, and should do so earlier rather than delaying.

The theme of help and health seeking in relation to FM assessment in pregnancy, based on high or moderate confidence in the findings, raises some concerns with regards to the support provided to women by healthcare professionals. This synthesis revealed that women were reluctant to contact healthcare professionals for fear of wasting their time, being burdensome, or being overly anxious. Findings of qualitative research exploring the culture of the NHS maternity services confirm that many women avoided imposing upon professionals by not accessing the services outside of their planned appointments, even when they were anxious or concerned [42]. Similarly, women indicated that healthcare professionals may minimally engage in discussions with FMs, or that the advice offered was often inconsistent, including variation in when to contact and what advice is offered when a woman makes contact reporting reduced FMs [43]. An international case (*n* = 153)-control (*n* = 480) study involving women who had experienced a stillbirth (within 30 days) compared to controls, showed that women who experienced a stillbirth were less likely to have been told by their health provider to monitor their FMs during pregnancy compared to healthy controls (adjusted Odds Ratio 0.55, 95% confidence interval 0.36–0.86, *p* = 0.008) [44]. In a further study, only 40% of women indicated that they felt happy with care/communication in talking to their care provider about their concerns [35]. This may cause women to resort predominantly to the internet or friends and family for information, rather than relying on healthcare professionals. A recent systematic review has shown that the quality of information regarding reduced FMs on the internet is widely varied [45]. This is concerning as it risks women accepting and relying on poor quality information, or misinformation. This synthesis reveals deficits in the provision of holistic, supportive care for women who are concerned for their baby’s wellbeing. There is a responsible onus on all maternity care providers to be attentive to women’s concerns, to support them in addressing their concerns and to reassure them, irrespective of the outcome, that their course of action in reporting their concerns was the correct one to take.

### Strengths and limitations

There are strengths and limitations to this synthesis that need to be acknowledged. Although the data contributing to the synthesis was explicitly qualitative, much of the data came from open-ended questions in survey studies. While these data provided valuable insights from the perspectives of women, due to the nature of survey design, the data are limited in providing depth in quality and quantity. Additionally, the aims of the included studies were varied, focusing on exclusive aspects of FM assessment in pregnancy, such as kick-charts [31], maternal hunger and satiation [29], and reduced FMs [37]. This resulted in studies providing data that were more focused on discrete aspects of FMs, because of the questions asked, rather than broadly exploring FM assessment from the perspectives of women. The included studies, however, were, for the most-part of high methodological quality. The majority of the findings were also assessed as having high or moderate confidence. This provides a degree of certainty that the emergent themes and the findings of this synthesis are truly reflective of women’s views, experiences and perceptions of assessing their FMs in pregnancy.

## Conclusion

This qualitative evidence synthesis reveals that women formally and informally engage with FMs in pregnancy. Women commonly adopt strategies to stimulate FMs if concerned and have awareness that the characteristics of FMs change as pregnancy progresses. Women, however, should be better supported by healthcare professionals in making contact should they have concerns for their FMs, and should be reassured of this course of action. Women should be advised, rather than spending time on non-evidence-based strategies to illicit FMs, to contact their healthcare provider with their concerns without delay. Furthermore, there is a need for greater understanding and healthcare provider knowledge surrounding FMs in pregnancy so that information provided to women is evidence-based and consistent. We identified three studies only that included women of at least 20 weeks gestation that were exclusively qualitative in design. To increase our depth and understanding of assessing FMs in pregnancy from the perspectives of women, additional qualitative studies are required.

## Supplementary Information

**Additional file 1.** ENTREQ Checklist. Enhancing transparency in reporting the synthesis of qualitative research, reporting checklist.

**Additional file 2.** Data Extraction Form (example).

**Additional file 3.** Table of Excluded Studies.

**Additional file 4.** Audit Trail of data synthesis.

**Additional file 5.** Evidence Profile (GRADE-CERQual).

## Data Availability

All data are available in the manuscript or in the Additional files. The corresponding author can be contacted for additional information if required.

## Abbreviations

AFFIRM: Awareness of fetal movements and care package to reduce fetal mortality
ANC: Antenatal clinic
ENTREQ: Enhancing Transparency in Reporting the synthesis of Qualitative research
EPPI: Evidence for Policy and Practice Information
FMs: Fetal Movements
GRADE-CERQual: Grading of Recommendations Assessment, Development and Evaluation-Confidence in the Evidence from Reviews of Qualitative research
HCP: Healthcare professional
PROSPERO: International Prospective Register of Systematic Reviews
RCOG: Royal College of Obstetricians and Gynaecologists
RFM: Reduced fetal movement
