# Supplementary material for: Assessing fetal movements in pregnancy: A qualitative evidence synthesis of women’s views, perspectives and experiences
Source: BMC Pregnancy Childbirth. 2021 Mar 10;21:197. doi: 10.1186/s12884-021-03667-y (PMC7944914; doi:10.1186/s12884-021-03667-y)
Supplement: Supplementary file 1 — Additional file 1. ENTREQ Checklist. Enhancing transparency in reporting the synthesis of qualitative research, reporting checklist. [file 12884_2021_3667_MOESM1_ESM.docx]

**Additional File 1: the ENTREQ Statement [Tong *et al.* 2012]**

| **No** | **Item** | **Guide and description** | **Page no. in manuscript** |
| --- | --- | --- | --- |
| 1 | Aim | State the research question the synthesis addresses. | p.5 |
| 2 | Synthesis methodology | Identify the synthesis methodology or theoretical framework which underpins the synthesis, and describe the rationale for choice of methodology *(e.g. meta-ethnography, thematic synthesis, critical interpretive synthesis, grounded theory synthesis, realist synthesis, meta-aggregation, meta-study, framework synthesis).* | p.5 |
| 3 | Approach to searching | Indicate whether the search was pre-planned (*comprehensive search strategies to seek all available studies)* or iterative (*to seek all available concepts until they theoretical saturation is achieved)*. | p.6 |
| 4 | Inclusion criteria | Specify the inclusion/exclusion criteria *(e.g. in terms of population, language, year limits, type of publication, study type).* | p.5 |
| 5 | Data sources | Describe the information sources used (e.g. *electronic databases (MEDLINE, EMBASE, CINAHL, psycINFO, Econlit), grey literature databases (digital thesis, policy reports), relevant organisational websites, experts, information specialists, generic web searches (Google Scholar) hand searching, reference lists)* and when the searches conducted; provide the rationale for using the data sources. | p.6 |
| 6 | Electronic Search strategy | Describe the literature search *(e.g. provide electronic search strategies with population terms, clinical or health topic terms, experiential or social phenomena related terms, filters for qualitative research, and search limits)*. | p.6 |
| 7 | Study screening methods | Describe the process of study screening and sifting *(e.g. title, abstract and full text review, number of independent reviewers who screened studies).* | p.6 |
| 8 | Study characteristics | Present the characteristics of the included studies *(e.g. year of publication, country, population, number of participants, data collection, methodology, analysis, research questions).* | p.10  Table 2 |
| 9 | Study selection results | Identify the number of studies screened and provide reasons for study exclusion *(e,g, for comprehensive searching, provide numbers of studies screened and reasons for exclusion indicated in a figure/flowchart; for iterative searching describe reasons for study exclusion and inclusion based on modifications t the research question and/or contribution to theory development).* | p.6  Figure 1  Additional File 1 |
| 10 | Rationale for appraisal | Describe the rationale and approach used to appraise the included studies or selected findings *(e.g. assessment of conduct (validity and robustness), assessment of reporting (transparency), and assessment of content and utility of the findings).* | p.7  Table 1 |
| 11 | Appraisal items | State the tools, frameworks and criteria used to appraise the studies or selected findings *(e.g. Existing tools: CASP, QARI, COREQ, Mays and Pope [25]; reviewer developed tools; describe the domains assessed: research team, study design, data analysis and interpretations, reporting).* | p.7  Table 1 |
| 12 | Appraisal process | Indicate whether the appraisal was conducted independently by more than one reviewer and if consensus was required. | p.7 |
| 13 | Appraisal results | Present results of the quality assessment and indicate which articles, if any, were weighted/excluded based on the assessment and give the rationale. | p.14  Table 3 |
| 14 | Data extraction | Indicate which sections of the primary studies were analysed and how were the data extracted from the primary studies? *(e.g. all text under the headings “results /conclusions” were extracted electronically and entered into a computer software).* | p.7-8 |
| 15 | Software | State the computer software used, if any. | N/A |
| 16 | Number of reviewers | Identify who was involved in coding and analysis. | p.7-8 |
| 17 | Coding | Describe the process for coding of data *(e.g. line by line coding to search for concepts).* | p.7-8  Additional File 2 |
| 18 | Study comparison | Describe how were comparisons made within and across studies *(e.g. subsequent studies were coded into pre-existing concepts, and new concepts were created when deemed necessary).* | p.7-8  Table 4 |
| 19 | Derivation of themes | Explain whether the process of deriving the themes or constructs was inductive or deductive. | p.7-8 |
| 20 | Quotations | Provide quotations from the primary studies to illustrate themes/constructs, and identify whether the quotations were participant quotations of the author’s interpretation. | p.16-24 |
| 21 | Synthesis output | Present rich, compelling and useful results that go beyond a summary of the primary studies (e.g. *new interpretation, models of evidence, conceptual models, analytical framework, development of a new theory or construct).* | p.16-24 |
