# Supplementary material for: Assessing fetal movements in pregnancy: A qualitative evidence synthesis of women’s views, perspectives and experiences
Source: BMC Pregnancy Childbirth. 2021 Mar 10;21:197. doi: 10.1186/s12884-021-03667-y (PMC7944914; doi:10.1186/s12884-021-03667-y)
Supplement: Supplementary file 2 — Additional file 2. Data Extraction Form (example). [file 12884_2021_3667_MOESM2_ESM.docx]

**Data Extraction Form: QES Review**

| Study Reference: | | |
| --- | --- | --- |
| Person extracting data: | Date data extracted: | |
| Aim of Study: | | |
| Study design: | | |
| Study setting/location(s): | Study period (dates): | |
| Sampling method (e.g. purposive, convenient, etc.): | | |
| Inclusion and Exclusion criteria: | | |
| Final numbers included in the study: | | |
| Description of the recruited sample (e.g. demographics, etc.) | | |
| Description of data collection method (e.g. questionnaire, interviews, location of data collection, etc.) | | |
| Description of data analysis method (e.g. content analysis, thematic, statistical, etc.): | | |
| **Findings:** Extract all data (including participant quotes) that you think reflects views or experiences of the phenomenon under investigation | | Location/ page in text |
|  | |  |
