# Supplementary material for: Assessing fetal movements in pregnancy: A qualitative evidence synthesis of women’s views, perspectives and experiences
Source: BMC Pregnancy Childbirth. 2021 Mar 10;21:197. doi: 10.1186/s12884-021-03667-y (PMC7944914; doi:10.1186/s12884-021-03667-y)
Supplement: Supplementary file 3 — Additional file 3. Table of Excluded Studies. [file 12884_2021_3667_MOESM3_ESM.docx]

**Additional File 3: Excluded studies with reasons**

| **Reference** | **Reason for excluding** |
| --- | --- |
| Rådestad I. Mindfetalness a method for structured observation on fetal movements. Women and Birth (Abstracts), 2018;30(S1): 1-47 | Conference abstract |
| Raynes-Greenow CH, Gordon A, Li Q, Bond D, Jones R, Hyett J, Ogle R. 561 Defining normal: qualitative descriptions of fetal movement in a normal healthy pregnant population (Conference abstract 561). Am J Epidemiol. 2011;173(Suppl):S141 | Conference abstract |
| Chan A. Hughes S. Service evaluation of antenatal guidance for monitoring fetal movements: effects on maternal understanding and reassurance (oral presentation 0031). BJOG 2014;125:9 | Conference abstract |
| Warland J. Heazell AEP. Stacey T. Coomarasamy C. Budd J. Mitchell EA. O'Brien LM. Reduced fetal movements: Should we use 'altered fetal activity' instead? Journal of Paediatrics and Child Health, 2018;54:51 | Conference abstract |
| Malm MC. Radestad I. Rubertsson C. Hildingsson I. Lindgren H. Women's experiences of two different self-assessment methods for monitoring fetal movements in full-term pregnancy--a crossover trial. BMC Pregnancy Childbirth, 2014;14:349 | Cross-over trial |
| Gordon A. Raynes-Greenow CH. Bond D. Jones R. Morris JM. Jeffery HE. Maternal perception of fetal movements: Quality vs quantity? The Sydney stillbirth study. Journal of Paediatrics and Child Health, 2011; 47:77 | Duplicate |
| Freda MC. Mikhail M. Mazloom E. Polizzotto R. Damus K. Merkatz I. Fetal movement counting: which method? MCN Am J Matern Child Nurs, 1993;18(6):314-312 | Duplicate |
| Dickinson R. Women's views on keeping fetal movement charts. Br J Obstet Gynaecol, 1986;93(10);1111-1112 | Letter to the Editor |
| Elbourne D. Grant A. Study results vary in count-to-10 method of fetal movement screening. Am J Obstet Gynecol, 1990;163(pt1):264-265 | Letter to the Editor |
| Matsubara S. Matsubara D. Takahashi H. Baba Y. Perceived fetal movement count as a traditional but essential tool for evaluating fetal health: some additions and Japanese obstetricians' and pediatricians' view. J Matern Fetal Neonatal Med, 2018;31(2):251-252 | Letter to the Editor |
| Thompson SL. Wheeler T. Compliance and maternal fetal movement counting. Lancet, 1985;2(8464):1122 | Letter to the Editor |
| Li YT. Wang PH. Perception of fetal movement in the pregnant women. J Chin Med Assoc, 2018;81(10):930-931 | Letter to the Editor |
| Torkestani F. Zafarghandi N. Davati A. Hadavand S. Farzinmoghadam S. Nasiri Z. The value of reporting decreased or absent fetal movements by mothers in predicting the pregnancy outcome. Iranian Journal of Medical Sciences, 2011;36(3):235-236 | Letter to the Editor |
| Davis L. Daily fetal movement counting. A valuable assessment tool. J Nurse Midwifery, 1987;32(1) | Literature review |
| Fardy HJ. The use of fetal kick charts. Aust Fam Physician, 1990;19(10):1565-1567. | Literature review |
| Grice R. Hobbs L. A literature review: how do women perceive fetal movements? MIDIRS Midwifery Digest, 2018;28(1):49-54 | Literature review |
| Baston H. Monitoring fetal wellbeing during routine antenatal care. Practising Midwife, 2003;6(4):29-33 | Literature review |
| Copper T. Wickham S. Evolution of the Theses: Monitoring fetal movement to assess well-being. Essentially MIDIRS, 2013;4(6):32-39 | Literature review |
| Marnoch A. An evaluation of the importance of formal, maternal fetal movement counting as a measure of fetal well-being. Midwifery, 1992;8(2):54-63 | Literature review |
| McNay MB. Fetal movements. Dev Med Child Neurol, 1988;30(6):821-824 | Literature review |
| Clark J. Britton K. Factors contributing to client nonuse of the Cardiff Count-to-ten fetal activity chart. J Nurse Midwifery, 1985;30(6):320-326 | No qualitative data |
| Eggertsen SC. Benedetti TJ. Maternal response to daily fetal movement counting in primary care settings. Am J Perinatol, 1987;4(4):327-330 | No qualitative data |
| Erlandsson K. Lindgren H Davidsson-Bremborg A. Radestad I. Women's premonitions prior to the death of their baby in utero and how they deal with the feeling that their baby may be unwell. Acta Obstet Gynecol Scand, 2012;91(1):28-33 | No qualitative data |
| Flenady V. Ellwood D. Bradford B. Coory M. Middleton P. Gardener G. Radestad I. Homer C. Davies-Tuck M. Forster D. Gordon A. Groom K. Crowther C. Walker S. Foord C. Warland J. Murphy M. Said J. Boyle F. O'Donoghue K. Beyond the headlines: Fetal movement awareness is an important stillbirth prevention strategy. Women & Birth, 2019;32(1) | No qualitative data |
| Freda MC. Mikhail M. Mazloom E. Polizzotto R. Damus K. Merkatz I. Fetal movement counting: which method?... two well-known methods are studied in an inner-city population. MCN: The American Journal of Maternal Child Nursing, 1993;18(6):314-321 | No qualitative data |
| Gibby NW. Relationship between fetal movement charting and anxiety in low-risk pregnant women. J Nurse Midwifery, 1988;33(4):185-188 | No qualitative data |
| Gordon A. Raynes-Greenow CH. Bond D. Jones R. Morris, JM. Jeffery HE. The sydney stillbirth study: What is important when assessing maternal perception of fetal movements, quality vs quantity? American Journal of Epidemiology, 2011;173:S140 | No qualitative data |
| Linde A. Pettersson K. Radestad I. Women's Experiences of Fetal Movements before the Confirmation of Fetal Death--Contractions Misinterpreted as Fetal Movement. Birth, 2015;42(2):189-194 | No qualitative data |
| Malm MC. Hildingsson I. Rubertsson C. Radestad I. Lindgren H. Prenatal attachment and its association with foetal movement during pregnancy - A population based survey. Women Birth, 2016;29(6):482-486 | No qualitative data |
| Malm MC. Lindgren H. Rubertsson C. Hildingsson I. Radestad I. Development of a tool to evaluate fetal movements in full-term pregnancy. Sex Reprod Healthcare, 2014;5(1) | No qualitative data |
| Maputle MS. Mothiba MT. Mothers' knowledge of foetal movements monitoring during pregnancy in relation to perinatal outcome. Health SA Gesondheid, 2006;11(2):13-22 | No qualitative data |
| McArdle A. Gamble J. Flenady V. Toohill J. Creedy D. Women's knowledge of fetal movements: A survey of pregnant women. Journal of Paediatrics and Child Health, 2013;49:124-125 | No qualitative data |
| Mills K. Vlack S. Flenady V. Boyle F. Hammill J. Ibarra G. Ibiebele I. Kildea S. Kilroy K. Grant T. Robertson V. Roe Y. Toombs M. Watego S. Wild S. Wojcieszek A. 'Understanding fetal language-what's bubba trying to tell us?': Development of a culturally appropriate fetal movements brochure for aboriginal and torres strait islander women. Journal of Paediatrics and Child Health, 2015;51:72 | No qualitative data |
| Osefo N. The applicability of the daily fetal movement count (DFMC) as a monitor for fetal well-being in the Nigerian women. East Afr Med J, 1984;61(5):406-411 | No qualitative data |
| Pakenham S. Copeland A. Farine D. Kick-starting action: Canadian women's understanding of fetal movement guidelines. J Obstet Gynaecol Can, 2013;35(2):111-118 | No qualitative data |
| Peat AM. Stacey T. Cronin R. McCowan LM. Maternal knowledge of fetal movements in late pregnancy. Aust N Z J Obstet Gynaecol, 2012;52(5):445-449 | No qualitative data |
| Pimenta BS. Nomura RM. Nakamura MU. Moron AF. Maternal anxiety and fetal movement patterns in late pregnancy. J Matern Fetal Neonatal Med, 2016;29(12):2008-2012 | No qualitative data |
| Ross E. Locating the foetal subject: Uncertain entities and foetal viability in accounts of first-time pregnancy. Women's Studies International Forum, 2016;58:58-67 | No qualitative data |
| Sandall J. Rance S. McCourt C. Rayment J. Mackintosh N. Watson K. Carter W. Women's experiences of speaking up for safety during pregnancy, labour and birth. Archives of Disease in Childhood: Fetal and Neonatal Edition, 2012;97:A5 | No qualitative data |
| Shafi MI. Dover MS. Dyer CA. Byrne P. Constantine G. Luesley DM. Pictorial fetal movement charts in a multiracial antenatal clinic. BMJ, 1989;298(6689):1688 | No qualitative data |
| Sparling JW. Wilhelm IJ. MacLeod AM. Green SD. Katz VL. Blanchard GF Jr. Huntington GS. Aydlett LA. Developing a taxonomy of fetal movement: the first step in a longitudinal collaborative study. Physical & Occupational Therapy in Pediatrics, 1990;10(1):43-46 | No qualitative data |
| St James-Roberts I. Menon-Johansson P. Predicting infant crying from fetal movement data: an exploratory study. Early Hum Dev, 1999;54(1):55-62 | No qualitative data |
| Stainton MC. Parents' awareness of their unborn infant in the third trimester. Birth: Issues in Perinatal Care, 1990;17(2):92-96 | No qualitative data |
| Weller M. Daly L. Gardener G. Henry, S. Reinebrant HE. Warrilow K. Flenady V. My baby's movements: Women's feedback about a mobile application intervention aimed at raising awareness of fetal movements. Journal of Paediatrics and Child Health, 2018;53:123-124 | No qualitative data |
| Weller M. Gardener G. Henry S. Ellwood D. Daly L. Warrilow K. Boyle F. Flenady V. My Baby's Movements: Integration of a mobile phone application into the antenatal education toolkit | No qualitative data |
| Georgsson S. Linde A. Pettersson K. Nilsson R. Radestad I. To be taken seriously and receive rapid and adequate care - Womens' requests when they consult health care for reduced fetal movements. Midwifery, 2016;40:102-108 | No qualitative data |
| Ghidini A. Newman AM. Cacace M. Han S. Chongasing K. Maternal perception of fetal movements near term: The problem of maternal compliance. Obstet Gynecol, 2018;131:171S | No qualitative data |
| Modzelewski A. East C. Women's experiences with fetal movements research. Journal of Paediatrics and Child Health, 2012; 28:110 | No qualitative data |
| Martinius J. Intrauterine fetal movements and emotional expression. Padiatrische Praxis, 2003;63(4):595-596 | Not in English |
| Rerod A. Maternal instinct in women with complicated pregnancy. Ginekol Pol, 1995;66(1):13-18 | Not in English |
| Pollock D. Warland J. Ziaian T. Pearson E. Cooper M. Breaking through the silence in antenatal care: Fetal movement and stillbirth education. Journal of Paediatrics and Child Health, 2018;54:43-44 | Conference abstract of included study |
| Raynes-Greenow C. Gordon A. Qiushuang Li. Hyett J. Examining maternal perception of fetal movements using a qualitative framework: it’s the type of movement rather than the number that may be important (poster 069). Journal of Paediatrics and Child Health, 2012;48(Suppl. 1):82–154 | Poster abstract of included study |
| Smith CV. Davis, SA. Rayburn WF. Patients' acceptance of monitoring fetal movement. A randomized comparison of charting techniques. J Reprod Med, 1992;37(2):144-146 | Randomised trial |
| Liston RM. Bloom K. Zimmer P. The psychological effects of counting fetal movements. Birth, 1994;21(3):135-140 | Randomised trial |
| Bohon C. von Nostitz P. 'I feel a kick!'... fetal movement has long been considered one sign of a healthy pregnancy. Parents, 1994;69(10):57-58 | Unable to obtain full text |
| Ross E. Gestating bodies: sensing foetal movement in first-time pregnancy. Sociology of Health and Illness, 2019, 41;1, 95-111 | Women <20 weeks included |
