# Supplementary material for: Assessing fetal movements in pregnancy: A qualitative evidence synthesis of women’s views, perspectives and experiences
Source: BMC Pregnancy Childbirth. 2021 Mar 10;21:197. doi: 10.1186/s12884-021-03667-y (PMC7944914; doi:10.1186/s12884-021-03667-y)
Supplement: Supplementary file 4 — Additional file 4. Audit Trail of data synthesis. [file 12884_2021_3667_MOESM4_ESM.docx]

**Additional File 2: Development of analytical themes**

| **Example codes** | **Descriptive themes** | **Analytical sub-themes** | **Analytical main themes** |
| --- | --- | --- | --- |
| FM chart causes worry;  Gives reassurance – know their babies were kicking; Unreliable guide to babies wellbeing;  No time;  Questioning value of chart;  Difficult to remember to complete chart; | Use of charts for assessing FMs | Formal engagement with FMs | How women engage with FMs |
| Patterns reassuring;  Recognition of FMs;  Influences on FM;  Experiencing first FMs;  FMs as means of communicating with baby | Subjective awareness and assessment | Informal engagement with FMs |  |
| Eliciting movement;  Stimulate movement with various means; Adopted strategies to encourage FMs | Encouraging FMs | Strategies to stimulate FMs |  |
| Expectations for FMs;  First FMs,  FM near term;  Slower and stronger towards term;  Sensations of FMs; | Describing how FMs feel, and character of FMs | Sensations associated with FMs | *‘…like a feather inside my belly’* **-** articulating and describing FMs |
| Timing varied during day;  FMs more common in evening;  Change in intensity;  Deviation from normal;  Change in pattern;  Pattern individual | Describing when FMs are felt and individualistic nature of FMs | Timing and frequency of FMs |  |
| Written information desirable;  Internet a commonly used source;  Only need information if concerned;  Too much info can cause anxiety;  Info from friends and family | Sourcing information on FMs | Information sources | FMs and help/health seeking |
| Worry if baby not active;  Feeling burdensome if contact HCP with concern;  Negative experience of contacting HCPs;  Reason for delay in attending HCP;  Conflicting information; | Communications with HCPs – problematic for some women | Interacting with healthcare professionals |  |
