# Supplementary material for: Assessing fetal movements in pregnancy: A qualitative evidence synthesis of women’s views, perspectives and experiences
Source: BMC Pregnancy Childbirth. 2021 Mar 10;21:197. doi: 10.1186/s12884-021-03667-y (PMC7944914; doi:10.1186/s12884-021-03667-y)
Supplement: Supplementary file 5 — Additional file 5. Evidence Profile (GRADE-CERQual). [file 12884_2021_3667_MOESM5_ESM.docx]

**Additional File 4: Evidence Profile - GRADE CERQual**

| **Finding** | **Contributing reports** | **Methodological limitations;** *quality of the studies which are contributing to finding* | **Coherence;** *extent of support for review finding from the underlying data* | **Adequacy;** *richness of data; depth and quantity contributing to the finding; includes design* | **Relevance; i***nclusion criteria for studies should closely mirror the review question; study aim, population and focused aspect of FM that is explored considered* | **Overall Confidence** |
| --- | --- | --- | --- | --- | --- | --- |
| **Analytical theme: *How women engage with FMs*** | | | | | | |
| Women identified perceived factors that impact FMs such as; mother’s position, time of day, and mother’s hunger/eating patterns | 34-39,43 | **No or very minor concerns**; most contributing studies were of high quality | **Minor concerns**; the data were more varied than this, but a number of examples from across the studies are included in the finding which (in-part) addresses this | **Minor concerns**; contributing studies provided equitable quantities of data and in fairly equal depth; with a 4/3 split of pure qualitative to survey designs | **Moderate concerns**; in general the contributing studies’ aims were on more focused aspects of FMs and these were quite varied (e.g. response to maternal hunger [35-36], aspects related to ANC [37], advice [43], etc.); but the emerging data, in general, aligns with explorations of women’s views of assessing FMs in pregnancy | **Moderate;** downgraded x 1 as moderate concerns on relevance and minor concerns on coherence and adequacy |
| Women associated FMs with health; regular, individualised patterns of FMs were viewed as reassuring and altered patterns as a cause for concern | 36-39,41,43 | **No or very minor concerns**; most contributing studies were of high quality | **No or very minor concerns**; this finding is consistent across the studies | **Minor concerns;** contributing studies provided equitable quantities of data and in fairly equal depth but only 2 of the 6 studies were pure qualitative in design | **Moderate concerns**; the contributing studies’ aims were diverse (e.g. views of completing FM charts [37], reduced FMs [38-39], advice [43], etc.) they have a general alignment with exploring women’s views of assessing FMs in pregnancy | **High**; not downgraded as moderate concerns on relevance only, minor concerns on adequacy but no concerns on coherence and methodological limitations |
| Informal monitoring of FMs acted as a mechanism of communication between mother and baby | 34-36 | **No or very minor concerns**; contributing studies were of moderate to high quality | **No or very minor concerns;** this finding is consistent across the studies | **Minor concerns;** contributing equal amounts and depth of data, although study [34] used a questionnaire sufficient expansive qualitative data captured | **Minor concerns**; diverse aims which focused on Mindfetalness and normal fetal activity as perceived by women but still well aligned with the review question of women’s views of assessing FMs in pregnancy; 1 study (2 reports) however, included nulliparous women only and of low risk [35-36] | **High;** not downgraded as minor or no concerns across all four CERQual components |
| Formal engagement with and assessment of FMs can cause worry and anxiety, but was also considered important, providing reassurances that the baby was well | 34,37,41 | **Serious concerns**; the contributing data from two studies [34,37] met 5 or less of the 12 quality criteria | **Moderate concerns;** the data were varied for this finding | **Moderate concerns**; all of the studies were surveys with open ended response options and study [37] contributed most of the data to this finding | **Minor concerns**; although the contributing studies had varied aims, in general, they had a focus on women’s views of formal /structured assessment of FMs other than [41] which focused on knowledge of FMs | **Very low**; downgraded x 3 due to serious concerns on methodological limitations, moderate concerns on coherence and adequacy, and minor concerns on relevance |
| When women were experiencing reduced or altered FMs, they adopted a variety of strategies to elicit movement | 36,38,39,41,43,44 | **No or very minor concerns;** most contributing studies were of high quality | **No or very minor concerns**; this finding is consistent across the studies, and strategies were varied | **Moderate concerns**; Most of the studies were surveys with open ended response options other than [36,44]; the quantity and depth of qualitative data provided by the studies was also varied | **Moderate concerns**; the contributing studies had varied aims, study [36] included nulliparous women only, and low risk, and study [44] included high risk women, i.e. those who had experienced reduced FMs | **Moderate;** downgraded x 1 due to moderate concerns on relevance and adequacy, but no concerns on coherence and methodological limitations |
| **Analytical theme: *Articulating and describing fetal movements*** | | | | | | |
| Women’s descriptions and sensations of FMs differed at different gestational ages with changes in FMs noted as pregnancy progressed | 35,46,41-43 | **No or very minor concerns**; most contributing studies were of high quality | **Minor concerns;** finding was fairly consistent across studies although descriptions were conflicting especially for early FMs | **Minor concerns**; the descriptions of data were rich, and of equal depth, although 3 of the 5 studies involved survey design, the qualitative data was relatively expansive | **Minor concerns**; although the contributing studies had varied aims, they were fairly well aligned to exploring women’s views and experiences; although study [36] included low risk nulliparous women only, and study [44] included women who had experienced reduced FMs | **High;** not downgraded as minor only or no concerns across all four CERQual components |
| Women’s expectation of the timing of first FMs and the frequency they experienced FMs throughout the day were varied | 35,37,41,43 | **Minor concerns**; due to low quality of study [4], but all 3 other studies were of high quality | **No or very minor concerns;** the varied findings were fairly consistent across studies | **Moderate concerns**; varied contributions from studies, and data superficial overall, with varied depth and quantity | **Moderate concerns**; the contributing studies had varied aims which were moderately aligned to exploring women’s views and experiences; study [36] included low risk nulliparous women only | **Low;** downgraded x 2 as moderate concerns for relevance and adequacy, and minor concerns for methodological limitations |
| Women commonly experienced increased FMs in the evening and before mealtimes | 36,37 | **Moderate concerns**; due to low quality of study [37] and only one other study contributing to the finding | **No or very minor concerns;** the finding was consistent across studies | **Moderate concerns**; data superficial overall, and of limited depth | **Minor concerns**; the contributing studies had varied aims but were aligned to exploring women’s views and experiences; however, study [36] included low risk nulliparous women only | **Low;** downgraded x 2 due to moderate concerns for methodological limitations and adequacy, and minor concerns for relevance |
| Women associated unusual or changed FMs with changes in frequency or absence of FMs, or changes in the sensation of FMs | 38,39,43 | **No or very minor concerns;** most contributing studies were of high quality | **No or very minor concerns;** the finding was consistent across studies | **Moderate concerns**; the descriptions of data were of equal depth, but not very rich and all 3 involved survey design | **Moderate concerns**; the aim of the studies and the populations were diverse (i.e. exploring normal FMs versus women who had reduced FMs) although the aims align fairly well with exploring experiences of assessing FMs in pregnancy | **Moderate;** downgraded x 1 due to moderate concerns for relevance and adequacy, and no concerns for coherence and methodological limitations |
| **Analytical theme: *Fetal movements and help/health seeking*** | | | | | | |
| Women accessed multiple information sources on FMs including; healthcare professionals, antenatal classes, books, the internet, and family and friends | 36,40,41,43,44 | **No or very minor concerns**; most contributing studies were of high quality | **No or very minor concerns**; finding consistent across studies | **Minor concerns;** contributing studies provided relatively equal amounts and depth of data, and although 3 of the studies used a questionnaire, sufficient expansive qualitative data were captured | **Minor concerns**;  although the contributing studies had varied aims, they were fairly well aligned to exploring women’s views and experiences; although study [36] included low risk nulliparous women only, and study [44] included women who had experienced reduced FMs | **High**; Not downgraded as minor only or no concerns across all four CERQual components |
| There were preferences towards receiving FM information particularly in the format of printed documentation such as a pamphlet or hand-out, although preferences for the types of information were mixed | 40,41,43 | **No or very minor concerns**; most contributing studies were of high quality | **Minor concerns;** finding was fairly consistent across studies although descriptions were conflicting in relation to preferences | **Minor concerns;** contributing studies provided relatively equal amounts and depth of data, although all 3 of the studies used a questionnaire with various quantities and depth of qualitative data captured | **Moderate concerns**; the aims of the contributing studies were quite varied, and only moderately aligned to exploring women’s views and experiences | **Moderate;** downgraded x 1 due to moderate concerns for relevance and minor concerns for adequacy and coherence |
| The internet was a common source of information often ahead of consulting a healthcare professional | 40,41,43,44 | **No or very minor concerns**; most contributing studies were of high quality | **No or very minor concerns**; finding was consistent across studies | **Minor concerns;** contributing studies provided relatively equal amounts and depth of data, although 3 of the 4 studies used a questionnaire with various quantities and depth of qualitative data captured | **Moderate concerns**; the aims of the contributing studies were quite varied, and mixed populations (women with RFM [44]) | **High;** not downgraded as moderate concerns for relevance and only minor concerns for adequacy |
| A decrease in FM was generally perceived as a cause for concern that warranted help from a healthcare professional | 36,37,39,41,43,44 | **No or very minor concerns**; most contributing studies were of high quality | **Minor concerns**; fairly consistent across studies, although diverse data in relation to FMs at term | **Moderate concerns;** data depth and quantity contributing to finding varied across studies with data largely derived from studies [39,44] | **Minor concerns**; the aims of the contributing studies were varied, but aligned fairly well to exploring views of FMs; but populations were mixed (women with RFM [39,44]) | **Moderate;** downgraded x 1 due to moderate concerns for adequacy, and minor concerns for relevance and coherence |
| Reasons for contacting healthcare professionals due to a decrease or change in FMs included; if a defined period of time had passed, if the worry became unmanageable, fear of fetal loss, unsuccessful strategies to stimulate FMs | 39,40,44 | **No or very minor concerns;** most contributing studies were of high quality | **No or very minor concerns**; finding was consistent across studies | **Minor concerns;** data depth and quantity contributing to finding relatively equal across studies | **Minor concerns**;  the aims of the contributing studies were varied, but aligned fairly well to exploring views of FMs; but populations were mixed (women with RFM [39,44]) | **High**; not downgraded as minor only or no concerns across all four CERQual components |
| Barriers to contacting healthcare professionals were mostly related to doubt or fear of being perceived a particular way, not being listened to, wasting healthcare professionals’ time | 39,41,43,44 | **No or very minor concerns;** most contributing studies were of high quality | **Minor concerns**; some contrasting (but limited) data in relation to attendance with concerns | **Moderate concerns;** data depth and quantity contributing to finding relatively equal across studies; although 3 of the 4 studies used a questionnaire with various quantities and depth of qualitative data captured | **Minor concerns**;  The aims of the contributing studies were varied, but aligned fairly well to exploring views of FMs; but populations were mixed (women with RFM [39,44]) | **High;** not downgraded as moderate concerns for adequacy only, and minor or no concerns in all other components |
| The advice offered by healthcare professionals to women on monitoring FMs and on what to do if they were concerned about FMs varied | 38-40,43,44 | **No or very minor concerns;** most contributing studies were of high quality | **No or very minor concerns**; finding was consistent across studies | **Moderate concerns;** data depth and quantity contributing to finding relatively equal across studies; although 3 of the 4 studies used a questionnaire with various quantities and depth of qualitative data captured | **Moderate concerns**;  The aims of the contributing studies were varied, especially [40] and were moderately aligned to exploring views of FMs; but populations were mixed (women with RFM [39,44]) | **Moderate;** downgrade x 1 due to moderate concerns for relevance and adequacy, but not concerns for coherence and methodological limitations |
